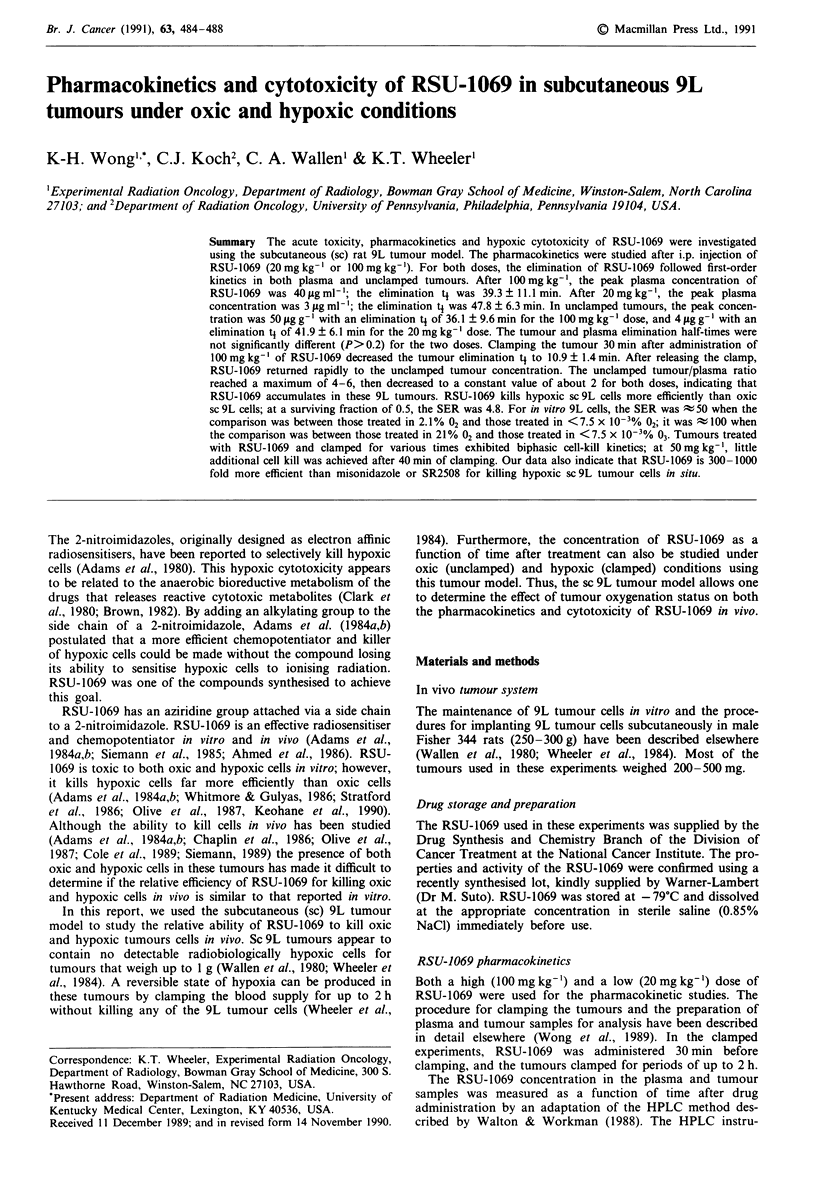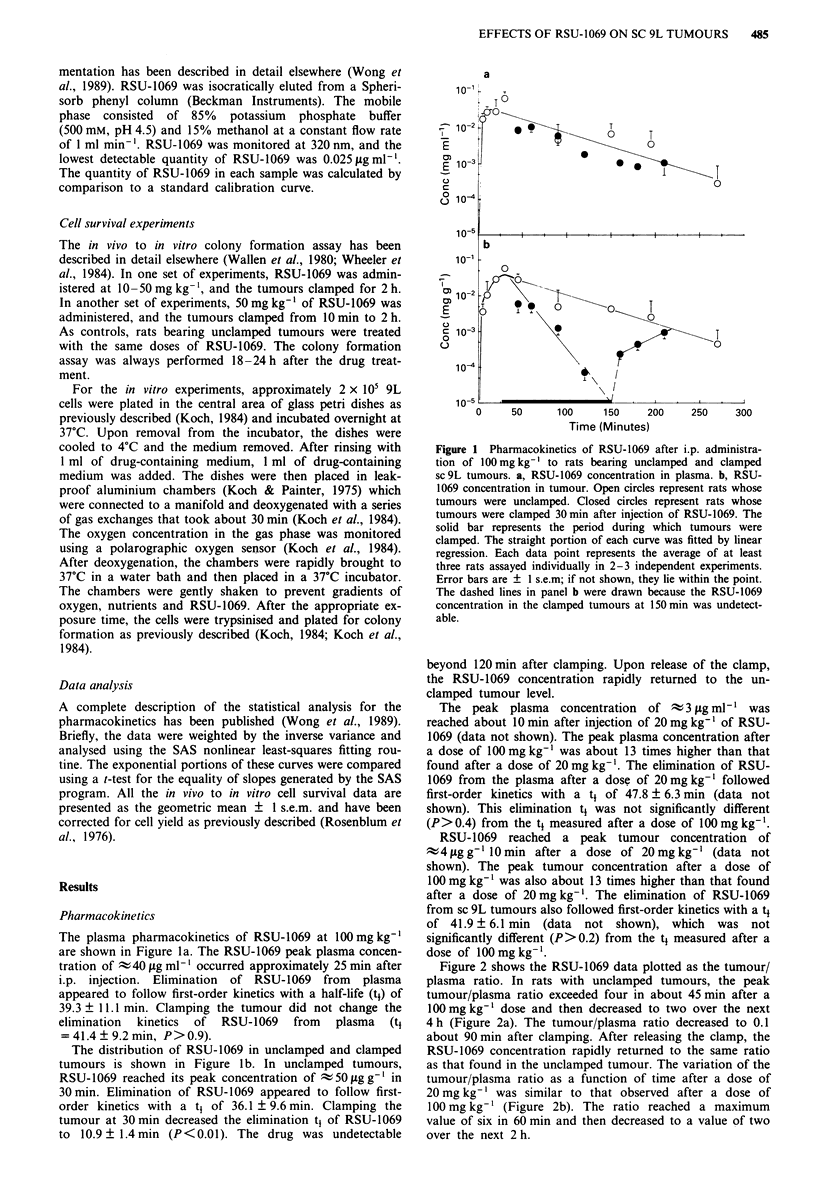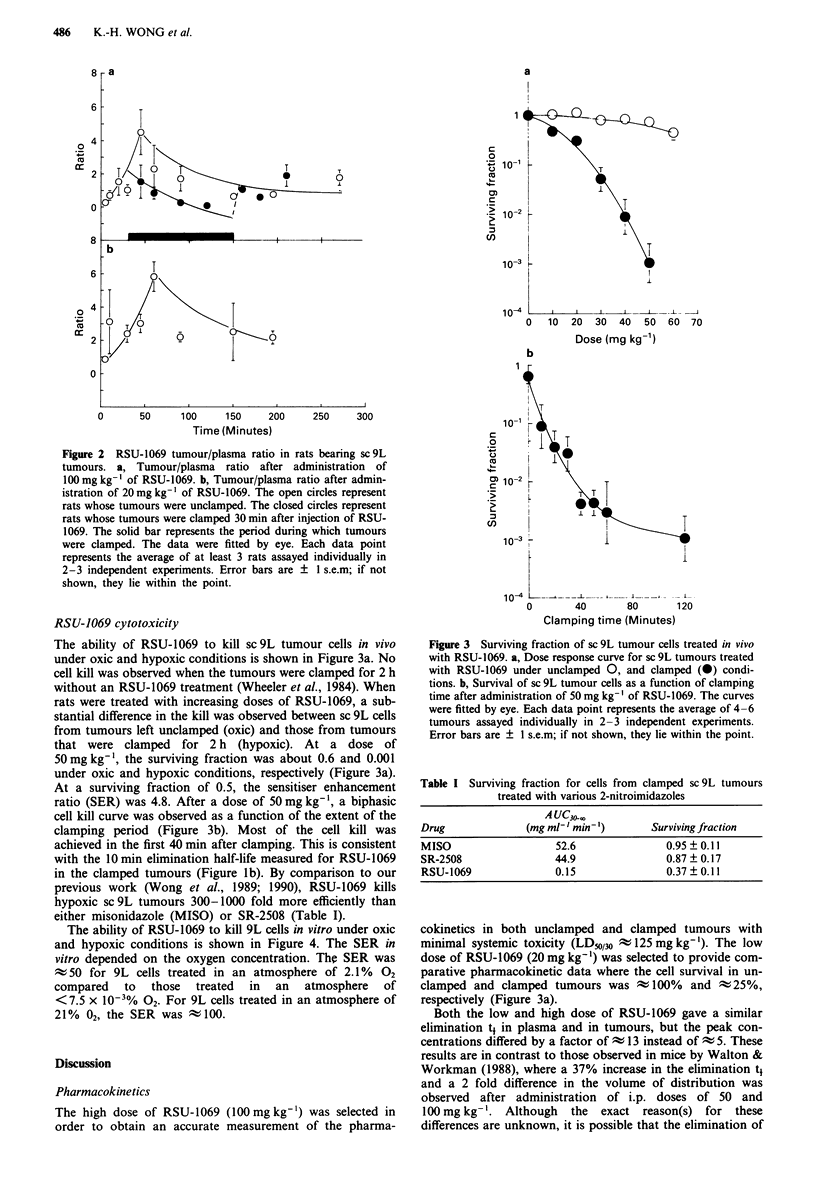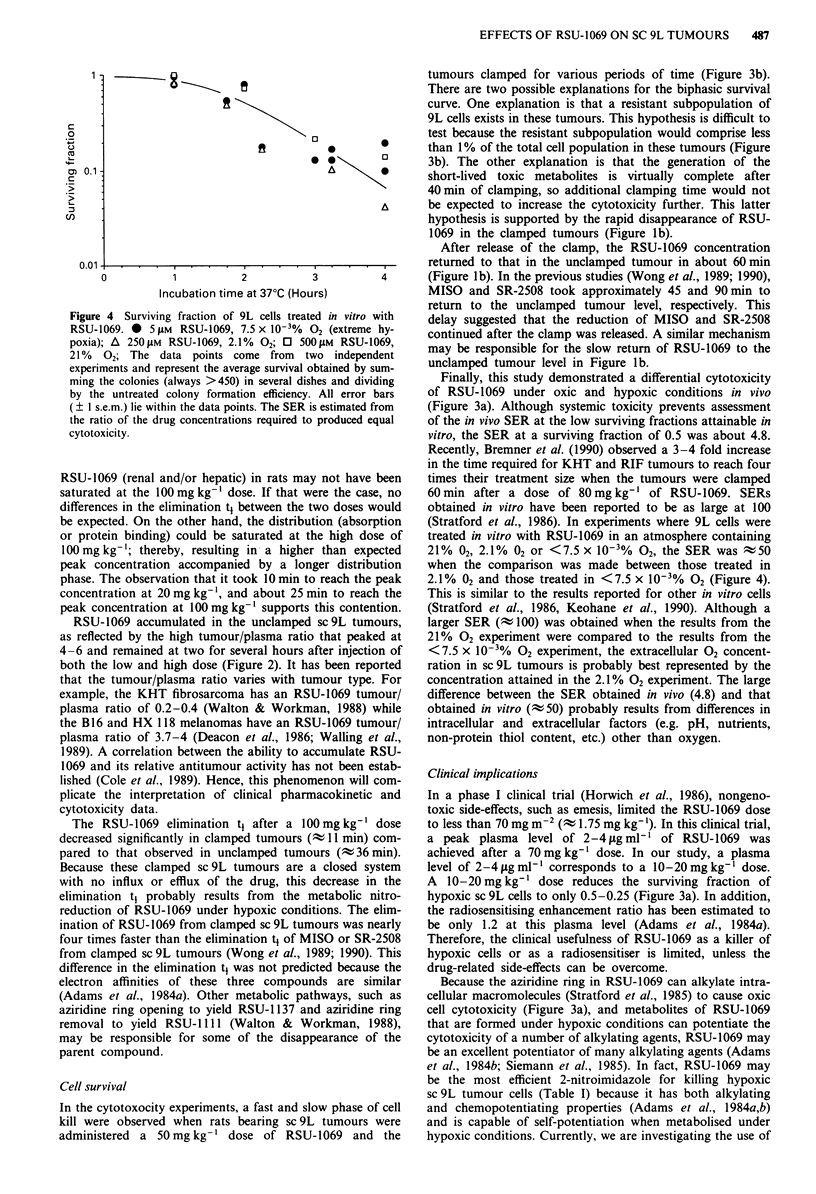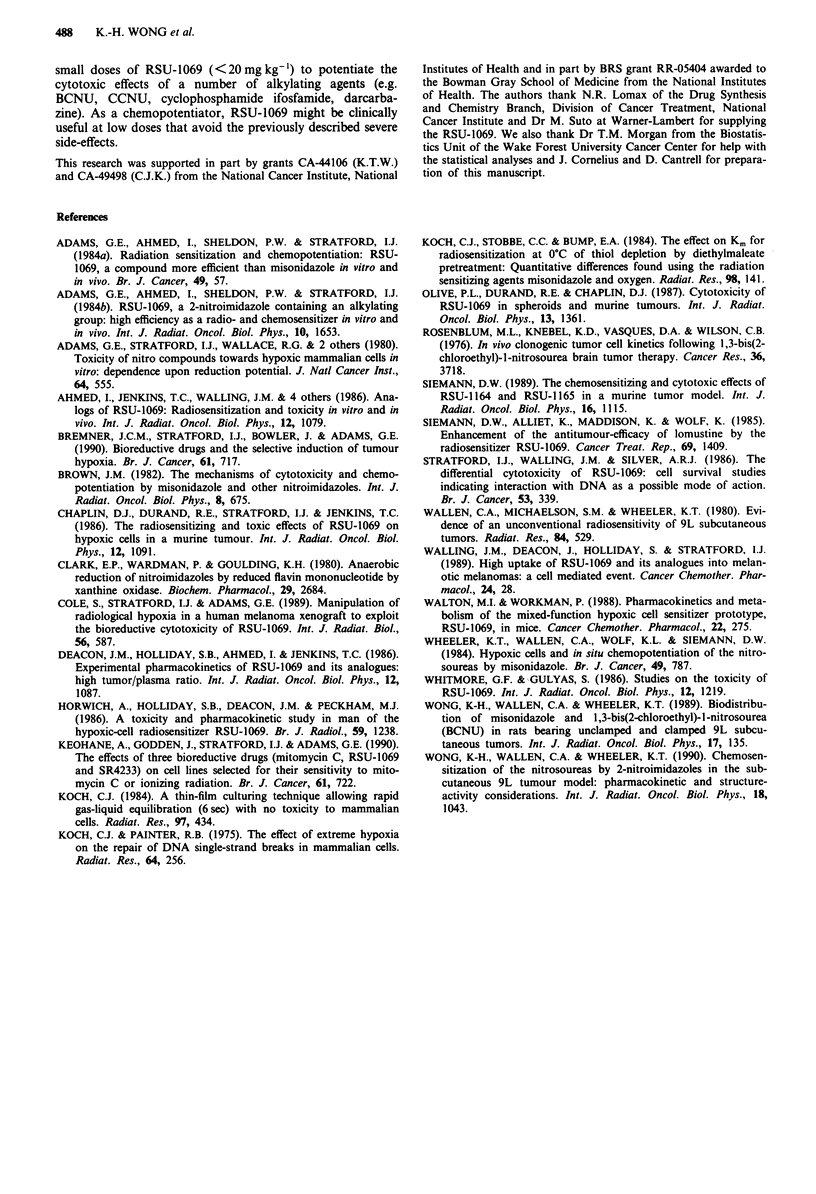# Pharmacokinetics and cytotoxicity of RSU-1069 in subcutaneous 9L tumours under oxic and hypoxic conditions.

**DOI:** 10.1038/bjc.1991.116

**Published:** 1991-04

**Authors:** K. H. Wong, C. J. Koch, C. A. Wallen, K. T. Wheeler

**Affiliations:** Department of Radiology, Bowman Gray School of Medicine, Winston-Salem, North Carolina 27103.

## Abstract

The acute toxicity, pharmacokinetics and hypoxic cytotoxicity of RSU-1069 were investigated using the subcutaneous (sc) rat 9L tumour model. The pharmacokinetics were studied after i.p. injection of RSU-1069 (20 mg kg-1 or 100 mg kg-1). For both doses, the elimination of RSU-1069 followed first-order kinetics in both plasma and unclamped tumours. After 100 mg kg-1, the peak plasma concentration of RSU-1069 was 40 micrograms ml-1; the elimination t1/2 was 39.3 +/- 11.1 min. After 20 mg kg-1, the peak plasma concentration was 3 micrograms ml-1; the elimination t1/2 was 47.8 +/- 6.3 min. In unclamped tumours, the peak concentration was 50 micrograms g-1 with an elimination t1/2 of 36.1 +/- 9.6 min for the 100 mg kg-1 dose, and 4 micrograms g-1 with an elimination t1/2 of 41.9 +/- 6.1 min for the 20 mg kg-1 dose. The tumour and plasma elimination half-times were not significantly different (P greater than 0.2) for the two doses. Clamping the tumour 30 min after administration of 100 mg kg-1 of RSU-1069 decreased the tumour elimination t1/2 to 10.9 +/- 1.4 min. After releasing the clamp, RSU-1069 returned rapidly to the unclamped tumour concentration. The unclamped tumour/plasma ratio reached a maximum of 4-6, then decreased to a constant value of about 2 for both doses, indicating that RSU-1069 accumulates in these 9L tumours. RSU-1069 kills hypoxic sc 9L cells more efficiently than oxic sc 9L cells; at a surviving fraction of 0.5, the SER was 4.8. For in vitro 9L cells, the SER was approximately 50 when the comparison was between those treated in 2.1% 0(2) and those treated in less than 7.5 x 10(-3)% 0(2); it was approximately 100 when the comparison was between those treated in 21% 0(2) and those treated in less than 7.5 x 10(-3)% 0(3). Tumours treated with RSU-1069 and clamped for various times exhibited biphasic cell-kill kinetics; at 50 mg kg-1, little additional cell kill was achieved after 40 min of clamping. Our data also indicate that RSU-1069 is 300-1000 fold more efficient than misonidazole or SR2508 for killing hypoxic sc 9L tumour cells in situ.


Br. J. Cancer (1991), 63, 484-488 © Macmillan Press Ltd., 1991

### Pharmacokinetics and cytotoxicity of RSU-1069 in subcutaneous 9L tumours under oxic and hypoxic conditions

K-H. Wong[1,*], C.J. Koch[2], C. A. Wallen[1] & K.T. Wheeler[1]

[1]*Experimental Radiation Oncology, Department of Radiology, Bowman Gray School of Medicine, Winston-Salem, North Carolina 27103; and* [2]*Department of Radiation Oncology, University of Pennsylvania, Philadelphia, Pennsylvania 19104, USA.*

**Summary** The acute toxicity, pharmacokinetics and hypoxic cytotoxicity of RSU-1069 were investigated using the subcutaneous (sc) rat 9L tumour model. The pharmacokinetics were studied after i.p. injection of RSU-1069 ($20 \text{ mg kg}^{-1}$ or $100 \text{ mg kg}^{-1}$). For both doses, the elimination of RSU-1069 followed first-order kinetics in both plasma and unclamped tumours. After $100 \text{ mg kg}^{-1}$, the peak plasma concentration of RSU-1069 was $40 \mu\text{g ml}^{-1}$; the elimination $t_{½}$ was $39.3 \pm 11.1$ min. After $20 \text{ mg kg}^{-1}$, the peak plasma concentration was $3 \mu\text{g ml}^{-1}$; the elimination $t_{½}$ was $47.8 \pm 6.3$ min. In unclamped tumours, the peak concentration was $50 \mu\text{g g}^{-1}$ with an elimination $t_{½}$ of $36.1 \pm 9.6$ min for the $100 \text{ mg kg}^{-1}$ dose, and $4 \mu\text{g g}^{-1}$ with an elimination $t_{½}$ of $41.9 \pm 6.1$ min for the $20 \text{ mg kg}^{-1}$ dose. The tumour and plasma elimination half-times were not significantly different ($P > 0.2$) for the two doses. Clamping the tumour 30 min after administration of $100 \text{ mg kg}^{-1}$ of RSU-1069 decreased the tumour elimination $t_{½}$ to $10.9 \pm 1.4$ min. After releasing the clamp, RSU-1069 returned rapidly to the unclamped tumour concentration. The unclamped tumour/plasma ratio reached a maximum of 4–6, then decreased to a constant value of about 2 for both doses, indicating that RSU-1069 accumulates in these 9L tumours. RSU-1069 kills hypoxic sc 9L cells more efficiently than oxic sc 9L cells; at a surviving fraction of 0.5, the SER was 4.8. For *in vitro* 9L cells, the SER was $\approx 50$ when the comparison was between those treated in 2.1% $0_2$ and those treated in $< 7.5 \times 10^{-3}\%$ $0_2$; it was $\approx 100$ when the comparison was between those treated in 21% $0_2$ and those treated in $< 7.5 \times 10^{-3}\%$ $0_3$. Tumours treated with RSU-1069 and clamped for various times exhibited biphasic cell-kill kinetics; at $50 \text{ mg kg}^{-1}$, little additional cell kill was achieved after 40 min of clamping. Our data also indicate that RSU-1069 is 300–1000 fold more efficient than misonidazole or SR2508 for killing hypoxic sc 9L tumour cells *in situ*.

The 2-nitroimidazoles, originally designed as electron affinic radiosensitisers, have been reported to selectively kill hypoxic cells (Adams *et al.*, 1980). This hypoxic cytotoxicity appears to be related to the anaerobic bioreductive metabolism of the drugs that releases reactive cytotoxic metabolites (Clark *et al.*, 1980; Brown, 1982). By adding an alkylating group to the side chain of a 2-nitroimidazole, Adams *et al.* (1984*a,b*) postulated that a more efficient chemopotentiator and killer of hypoxic cells could be made without the compound losing its ability to sensitise hypoxic cells to ionising radiation. RSU-1069 was one of the compounds synthesised to achieve this goal.

RSU-1069 has an aziridine group attached via a side chain to a 2-nitroimidazole. RSU-1069 is an effective radiosensitiser and chemopotentiator *in vitro* and *in vivo* (Adams *et al.*, 1984*a,b*; Siemann *et al.*, 1985; Ahmed *et al.*, 1986). RSU-1069 is toxic to both oxic and hypoxic cells *in vitro*; however, it kills hypoxic cells far more efficiently than oxic cells (Adams *et al.*, 1984*a,b*; Whitmore & Gulyas, 1986; Stratford *et al.*, 1986; Olive *et al.*, 1987, Keohane *et al.*, 1990). Although the ability to kill cells *in vivo* has been studied (Adams *et al.*, 1984*a,b*; Chaplin *et al.*, 1986; Olive *et al.*, 1987; Cole *et al.*, 1989; Siemann, 1989) the presence of both oxic and hypoxic cells in these tumours has made it difficult to determine if the relative efficiency of RSU-1069 for killing oxic and hypoxic cells *in vivo* is similar to that reported *in vitro*.

In this report, we used the subcutaneous (sc) 9L tumour model to study the relative ability of RSU-1069 to kill oxic and hypoxic tumours cells *in vivo*. Sc 9L tumours appear to contain no detectable radiobiologically hypoxic cells for tumours that weigh up to 1 g (Wallen *et al.*, 1980; Wheeler *et al.*, 1984). A reversible state of hypoxia can be produced in these tumours by clamping the blood supply for up to 2 h without killing any of the 9L tumour cells (Wheeler *et al.*, 1984). Furthermore, the concentration of RSU-1069 as a function of time after treatment can also be studied under oxic (unclamped) and hypoxic (clamped) conditions using this tumour model. Thus, the sc 9L tumour model allows one to determine the effect of tumour oxygenation status on both the pharmacokinetics and cytotoxicity of RSU-1069 *in vivo*.

### Materials and methods

### In vivo *tumour system*

The maintenance of 9L tumour cells *in vitro* and the procedures for implanting 9L tumour cells subcutaneously in male Fisher 344 rats (250–300 g) have been described elsewhere (Wallen *et al.*, 1980; Wheeler *et al.*, 1984). Most of the tumours used in these experiments weighed 200–500 mg.

### *Drug storage and preparation*

The RSU-1069 used in these experiments was supplied by the Drug Synthesis and Chemistry Branch of the Division of Cancer Treatment at the National Cancer Institute. The properties and activity of the RSU-1069 were confirmed using a recently synthesised lot, kindly supplied by Warner-Lambert (Dr M. Suto). RSU-1069 was stored at $-79°C$ and dissolved at the appropriate concentration in sterile saline (0.85% NaCl) immediately before use.

### *RSU-1069 pharmacokinetics*

Both a high ($100 \text{ mg kg}^{-1}$) and a low ($20 \text{ mg kg}^{-1}$) dose of RSU-1069 were used for the pharmacokinetic studies. The procedure for clamping the tumours and the preparation of plasma and tumour samples for analysis have been described in detail elsewhere (Wong *et al.*, 1989). In the clamped experiments, RSU-1069 was administered 30 min before clamping, and the tumours clamped for periods of up to 2 h.

The RSU-1069 concentration in the plasma and tumour samples was measured as a function of time after drug administration by an adaptation of the HPLC method described by Walton & Workman (1988). The HPLC instru-

Correspondence: K.T. Wheeler, Experimental Radiation Oncology, Department of Radiology, Bowman Gray School of Medicine, 300 S. Hawthorne Road, Winston-Salem, NC 27103, USA.
*Present address: Department of Radiation Medicine, University of Kentucky Medical Center, Lexington, KY 40536, USA.
Received 11 December 1989; and in revised form 14 November 1990.

mentation has been described in detail elsewhere (Wong *et al.*, 1989). RSU-1069 was isocratically eluted from a Spherisorb phenyl column (Beckman Instruments). The mobile phase consisted of 85% potassium phosphate buffer (500 mM, pH 4.5) and 15% methanol at a constant flow rate of 1 ml $min^{-1}$. RSU-1069 was monitored at 320 nm, and the lowest detectable quantity of RSU-1069 was 0.025 $\mu g\ ml^{-1}$. The quantity of RSU-1069 in each sample was calculated by comparison to a standard calibration curve.

### *Cell survival experiments*

The *in vivo* to *in vitro* colony formation assay has been described in detail elsewhere (Wallen *et al.*, 1980; Wheeler *et al.*, 1984). In one set of experiments, RSU-1069 was administered at 10–50 mg $kg^{-1}$, and the tumours clamped for 2 h. In another set of experiments, 50 mg $kg^{-1}$ of RSU-1069 was administered, and the tumours clamped from 10 min to 2 h. As controls, rats bearing unclamped tumours were treated with the same doses of RSU-1069. The colony formation assay was always performed 18–24 h after the drug treatment.

For the *in vitro* experiments, approximately $2 \times 10^5$ 9L cells were plated in the central area of glass petri dishes as previously described (Koch, 1984) and incubated overnight at 37°C. Upon removal from the incubator, the dishes were cooled to 4°C and the medium removed. After rinsing with 1 ml of drug-containing medium, 1 ml of drug-containing medium was added. The dishes were then placed in leak-proof aluminium chambers (Koch & Painter, 1975) which were connected to a manifold and deoxygenated with a series of gas exchanges that took about 30 min (Koch *et al.*, 1984). The oxygen concentration in the gas phase was monitored using a polarographic oxygen sensor (Koch *et al.*, 1984). After deoxygenation, the chambers were rapidly brought to 37°C in a water bath and then placed in a 37°C incubator. The chambers were gently shaken to prevent gradients of oxygen, nutrients and RSU-1069. After the appropriate exposure time, the cells were trypsinised and plated for colony formation as previously described (Koch, 1984; Koch *et al.*, 1984).

### *Data analysis*

A complete description of the statistical analysis for the pharmacokinetics has been published (Wong *et al.*, 1989). Briefly, the data were weighted by the inverse variance and analysed using the SAS nonlinear least-squares fitting routine. The exponential portions of these curves were compared using a *t*-test for the equality of slopes generated by the SAS program. All the *in vivo* to *in vitro* cell survival data are presented as the geometric mean ± 1 s.e.m. and have been corrected for cell yield as previously described (Rosenblum *et al.*, 1976).

### Results

### *Pharmacokinetics*

The plasma pharmacokinetics of RSU-1069 at 100 mg $kg^{-1}$ are shown in Figure 1a. The RSU-1069 peak plasma concentration of ≈40 $\mu g\ ml^{-1}$ occurred approximately 25 min after i.p. injection. Elimination of RSU-1069 from plasma appeared to follow first-order kinetics with a half-life ($t_{1/2}$) of 39.3 ± 11.1 min. Clamping the tumour did not change the elimination kinetics of RSU-1069 from plasma ($t_{1/2}$ = 41.4 ± 9.2 min, $P > 0.9$).

The distribution of RSU-1069 in unclamped and clamped tumours is shown in Figure 1b. In unclamped tumours, RSU-1069 reached its peak concentration of ≈50 $\mu g\ g^{-1}$ in 30 min. Elimination of RSU-1069 appeared to follow first-order kinetics with a $t_{1/2}$ of 36.1 ± 9.6 min. Clamping the tumour at 30 min decreased the elimination $t_{1/2}$ of RSU-1069 to 10.9 ± 1.4 min ($P < 0.01$). The drug was undetectable

**Figure 1** Pharmacokinetics of RSU-1069 after i.p. administration of 100 mg $kg^{-1}$ to rats bearing unclamped and clamped sc 9L tumours. **a**, RSU-1069 concentration in plasma. **b**, RSU-1069 concentration in tumour. Open circles represent rats whose tumours were unclamped. Closed circles represent rats whose tumours were clamped 30 min after injection of RSU-1069. The solid bar represents the period during which tumours were clamped. The straight portion of each curve was fitted by linear regression. Each data point represents the average of at least three rats assayed individually in 2–3 independent experiments. Error bars are ± 1 s.e.m; if not shown, they lie within the point. The dashed lines in panel **b** were drawn because the RSU-1069 concentration in the clamped tumours at 150 min was undetectable.

beyond 120 min after clamping. Upon release of the clamp, the RSU-1069 concentration rapidly returned to the unclamped tumour level.

The peak plasma concentration of ≈3 $\mu g\ ml^{-1}$ was reached about 10 min after injection of 20 mg $kg^{-1}$ of RSU-1069 (data not shown). The peak plasma concentration after a dose of 100 mg $kg^{-1}$ was about 13 times higher than that found after a dose of 20 mg $kg^{-1}$. The elimination of RSU-1069 from the plasma after a dose of 20 mg $kg^{-1}$ followed first-order kinetics with a $t_{1/2}$ of 47.8 ± 6.3 min (data not shown). This elimination $t_{1/2}$ was not significantly different ($P > 0.4$) from the $t_{1/2}$ measured after a dose of 100 mg $kg^{-1}$.

RSU-1069 reached a peak tumour concentration of ≈4 $\mu g\ g^{-1}$ 10 min after a dose of 20 mg $kg^{-1}$ (data not shown). The peak tumour concentration after a dose of 100 mg $kg^{-1}$ was also about 13 times higher than that found after a dose of 20 mg $kg^{-1}$. The elimination of RSU-1069 from sc 9L tumours also followed first-order kinetics with a $t_{1/2}$ of 41.9 ± 6.1 min (data not shown), which was not significantly different ($P > 0.2$) from the $t_{1/2}$ measured after a dose of 100 mg $kg^{-1}$.

Figure 2 shows the RSU-1069 data plotted as the tumour/plasma ratio. In rats with unclamped tumours, the peak tumour/plasma ratio exceeded four in about 45 min after a 100 mg $kg^{-1}$ dose and then decreased to two over the next 4 h (Figure 2a). The tumour/plasma ratio decreased to 0.1 about 90 min after clamping. After releasing the clamp, the RSU-1069 concentration rapidly returned to the same ratio as that found in the unclamped tumour. The variation of the tumour/plasma ratio as a function of time after a dose of 20 mg $kg^{-1}$ was similar to that observed after a dose of 100 mg $kg^{-1}$ (Figure 2b). The ratio reached a maximum value of six in 60 min and then decreased to a value of two over the next 2 h.

**Figure 2** RSU-1069 tumour/plasma ratio in rats bearing sc 9L tumours. **a**, Tumour/plasma ratio after administration of 100 mg $kg^{-1}$ of RSU-1069. **b**, Tumour/plasma ratio after administration of 20 mg $kg^{-1}$ of RSU-1069. The open circles represent rats whose tumours were unclamped. The closed circles represent rats whose tumours were clamped 30 min after injection of RSU-1069. The solid bar represents the period during which tumours were clamped. The data were fitted by eye. Each data point represents the average of at least 3 rats assayed individually in 2–3 independent experiments. Error bars are ± 1 s.e.m; if not shown, they lie within the point.

**Figure 3** Surviving fraction of sc 9L tumour cells treated *in vivo* with RSU-1069. **a**, Dose response curve for sc 9L tumours treated with RSU-1069 under unclamped O, and clamped (●) conditions. **b**, Survival of sc 9L tumour cells as a function of clamping time after administration of 50 mg $kg^{-1}$ of RSU-1069. The curves were fitted by eye. Each data point represents the average of 4–6 tumours assayed individually in 2–3 independent experiments. Error bars are ± 1 s.e.m; if not shown, they lie within the point.

*RSU-1069 cytotoxicity*

The ability of RSU-1069 to kill sc 9L tumour cells *in vivo* under oxic and hypoxic conditions is shown in Figure 3a. No cell kill was observed when the tumours were clamped for 2 h without an RSU-1069 treatment (Wheeler *et al.*, 1984). When rats were treated with increasing doses of RSU-1069, a substantial difference in the kill was observed between sc 9L cells from tumours left unclamped (oxic) and those from tumours that were clamped for 2 h (hypoxic). At a dose of 50 mg $kg^{-1}$, the surviving fraction was about 0.6 and 0.001 under oxic and hypoxic conditions, respectively (Figure 3a). At a surviving fraction of 0.5, the sensitiser enhancement ratio (SER) was 4.8. After a dose of 50 mg $kg^{-1}$, a biphasic cell kill curve was observed as a function of the extent of the clamping period (Figure 3b). Most of the cell kill was achieved in the first 40 min after clamping. This is consistent with the 10 min elimination half-life measured for RSU-1069 in the clamped tumours (Figure 1b). By comparison to our previous work (Wong *et al.*, 1989; 1990), RSU-1069 kills hypoxic sc 9L tumours 300–1000 fold more efficiently than either misonidazole (MISO) or SR-2508 (Table I).

The ability of RSU-1069 to kill 9L cells *in vitro* under oxic and hypoxic conditions is shown in Figure 4. The SER *in vitro* depended on the oxygen concentration. The SER was ≈50 for 9L cells treated in an atmosphere of 2.1% $O_2$ compared to those treated in an atmosphere of <7.5 × $10^{-3}$% $O_2$. For 9L cells treated in an atmosphere of 21% $0_2$, the SER was ≈100.

**Table I** Surviving fraction for cells from clamped sc 9L tumours treated with various 2-nitroimidazoles

| *Drug* | $AUC_{30-\infty}$ (*mg ml*$^{-1}$ *min*$^{-1}$) | *Surviving fraction* |
|---|---|---|
| MISO | 52.6 | 0.95 ± 0.11 |
| SR-2508 | 44.9 | 0.87 ± 0.17 |
| RSU-1069 | 0.15 | 0.37 ± 0.11 |

### Discussion

*Pharmacokinetics*

The high dose of RSU-1069 (100 mg $kg^{-1}$) was selected in order to obtain an accurate measurement of the pharmacokinetics in both unclamped and clamped tumours with minimal systemic toxicity ($LD_{50/30}$ ≈125 mg $kg^{-1}$). The low dose of RSU-1069 (20 mg $kg^{-1}$) was selected to provide comparative pharmacokinetic data where the cell survival in unclamped and clamped tumours was ≈100% and ≈25%, respectively (Figure 3a).

Both the low and high dose of RSU-1069 gave a similar elimination $t_{\frac{1}{2}}$ in plasma and in tumours, but the peak concentrations differed by a factor of ≈13 instead of ≈5. These results are in contrast to those observed in mice by Walton & Workman (1988), where a 37% increase in the elimination $t_{\frac{1}{2}}$ and a 2 fold difference in the volume of distribution was observed after administration of i.p. doses of 50 and 100 mg $kg^{-1}$. Although the exact reason(s) for these differences are unknown, it is possible that the elimination of

**Figure 4** Surviving fraction of 9L cells treated *in vitro* with RSU-1069. ● 5 μM RSU-1069, 7.5 × $10^{-3}$% $O_2$ (extreme hypoxia); Δ 250 μM RSU-1069, 2.1% $O_2$; □ 500 μM RSU-1069, 21% $O_2$; The data points come from two independent experiments and represent the average survival obtained by summing the colonies (always > 450) in several dishes and dividing by the untreated colony formation efficiency. All error bars (± 1 s.e.m.) lie within the data points. The SER is estimated from the ratio of the drug concentrations required to produced equal cytotoxicity.

RSU-1069 (renal and/or hepatic) in rats may not have been saturated at the 100 mg $kg^{-1}$ dose. If that were the case, no differences in the elimination $t_{\frac{1}{2}}$ between the two doses would be expected. On the other hand, the distribution (absorption or protein binding) could be saturated at the high dose of 100 mg $kg^{-1}$; thereby, resulting in a higher than expected peak concentration accompanied by a longer distribution phase. The observation that it took 10 min to reach the peak concentration at 20 mg $kg^{-1}$, and about 25 min to reach the peak concentration at 100 mg $kg^{-1}$ supports this contention.

RSU-1069 accumulated in the unclamped sc 9L tumours, as reflected by the high tumour/plasma ratio that peaked at 4–6 and remained at two for several hours after injection of both the low and high dose (Figure 2). It has been reported that the tumour/plasma ratio varies with tumour type. For example, the KHT fibrosarcoma has an RSU-1069 tumour/plasma ratio of 0.2–0.4 (Walton & Workman, 1988) while the B16 and HX 118 melanomas have an RSU-1069 tumour/plasma ratio of 3.7–4 (Deacon *et al.*, 1986; Walling *et al.*, 1989). A correlation between the ability to accumulate RSU-1069 and its relative antitumour activity has not been established (Cole *et al.*, 1989). Hence, this phenomenon will complicate the interpretation of clinical pharmacokinetic and cytotoxicity data.

The RSU-1069 elimination $t_{\frac{1}{2}}$ after a 100 mg $kg^{-1}$ dose decreased significantly in clamped tumours (≈11 min) compared to that observed in unclamped tumours (≈36 min). Because these clamped sc 9L tumours are a closed system with no influx or efflux of the drug, this decrease in the elimination $t_{\frac{1}{2}}$ probably results from the metabolic nitroreduction of RSU-1069 under hypoxic conditions. The elimination of RSU-1069 from clamped sc 9L tumours was nearly four times faster than the elimination $t_{\frac{1}{2}}$ of MISO or SR-2508 from clamped sc 9L tumours (Wong *et al.*, 1989; 1990). This difference in the elimination $t_{\frac{1}{2}}$ was not predicted because the electron affinities of these three compounds are similar (Adams *et al.*, 1984*a*). Other metabolic pathways, such as aziridine ring opening to yield RSU-1137 and aziridine ring removal to yield RSU-1111 (Walton & Workman, 1988), may be responsible for some of the disappearance of the parent compound.

### *Cell survival*

In the cytotoxocity experiments, a fast and slow phase of cell kill were observed when rats bearing sc 9L tumours were administered a 50 mg $kg^{-1}$ dose of RSU-1069 and the tumours clamped for various periods of time (Figure 3b). There are two possible explanations for the biphasic survival curve. One explanation is that a resistant subpopulation of 9L cells exists in these tumours. This hypothesis is difficult to test because the resistant subpopulation would comprise less than 1% of the total cell population in these tumours (Figure 3b). The other explanation is that the generation of the short-lived toxic metabolites is virtually complete after 40 min of clamping, so additional clamping time would not be expected to increase the cytotoxicity further. This latter hypothesis is supported by the rapid disappearance of RSU-1069 in the clamped tumours (Figure 1b).

After release of the clamp, the RSU-1069 concentration returned to that in the unclamped tumour in about 60 min (Figure 1b). In the previous studies (Wong *et al.*, 1989; 1990), MISO and SR-2508 took approximately 45 and 90 min to return to the unclamped tumour level, respectively. This delay suggested that the reduction of MISO and SR-2508 continued after the clamp was released. A similar mechanism may be responsible for the slow return of RSU-1069 to the unclamped tumour level in Figure 1b.

Finally, this study demonstrated a differential cytotoxicity of RSU-1069 under oxic and hypoxic conditions *in vivo* (Figure 3a). Although systemic toxicity prevents assessment of the *in vivo* SER at the low surviving fractions attainable *in vitro*, the SER at a surviving fraction of 0.5 was about 4.8. Recently, Bremner *et al.* (1990) observed a 3–4 fold increase in the time required for KHT and RIF tumours to reach four times their treatment size when the tumours were clamped 60 min after a dose of 80 mg $kg^{-1}$ of RSU-1069. SERs obtained *in vitro* have been reported to be as large at 100 (Stratford *et al.*, 1986). In experiments where 9L cells were treated *in vitro* with RSU-1069 in an atmosphere containing 21% $0_2$, 2.1% $0_2$ or < 7.5 × $10^{-3}$% $O_2$, the SER was ≈50 when the comparison was made between those treated in 2.1% $0_2$ and those treated in < 7.5 × $10^{-3}$% $O_2$ (Figure 4). This is similar to the results reported for other *in vitro* cells (Stratford *et al.*, 1986, Keohane *et al.*, 1990). Although a larger SER (≈100) was obtained when the results from the 21% $O_2$ experiment were compared to the results from the < 7.5 × $10^{-3}$% $O_2$ experiment, the extracellular $O_2$ concentration in sc 9L tumours is probably best represented by the concentration attained in the 2.1% $O_2$ experiment. The large difference between the SER obtained *in vivo* (4.8) and that obtained *in vitro* (≈50) probably results from differences in intracellular and extracellular factors (e.g. pH, nutrients, non-protein thiol content, etc.) other than oxygen.

### *Clinical implications*

In a phase I clinical trial (Horwich *et al.*, 1986), nongenotoxic side-effects, such as emesis, limited the RSU-1069 dose to less than 70 mg $m^{-2}$ (≈1.75 mg $kg^{-1}$). In this clinical trial, a peak plasma level of 2–4 μg $ml^{-1}$ of RSU-1069 was achieved after a 70 mg $kg^{-1}$ dose. In our study, a plasma level of 2–4 μg $ml^{-1}$ corresponds to a 10–20 mg $kg^{-1}$ dose. A 10–20 mg $kg^{-1}$ dose reduces the surviving fraction of hypoxic sc 9L cells to only 0.5–0.25 (Figure 3a). In addition, the radiosensitising enhancement ratio has been estimated to be only 1.2 at this plasma level (Adams *et al.*, 1984*a*). Therefore, the clinical usefulness of RSU-1069 as a killer of hypoxic cells or as a radiosensitiser is limited, unless the drug-related side-effects can be overcome.

Because the aziridine ring in RSU-1069 can alkylate intracellular macromolecules (Stratford *et al.*, 1985) to cause oxic cell cytotoxicity (Figure 3a), and metabolites of RSU-1069 that are formed under hypoxic conditions can potentiate the cytotoxicity of a number of alkylating agents, RSU-1069 may be an excellent potentiator of many alkylating agents (Adams *et al.*, 1984*b*; Siemann *et al.*, 1985). In fact, RSU-1069 may be the most efficient 2-nitroimidazole for killing hypoxic sc 9L tumour cells (Table I) because it has both alkylating and chemopotentiating properties (Adams *et al.*, 1984*a*,*b*) and is capable of self-potentiation when metabolised under hypoxic conditions. Currently, we are investigating the use of small doses of RSU-1069 (<20 mg kg$^{-1}$) to potentiate the cytotoxic effects of a number of alkylating agents (e.g. BCNU, CCNU, cyclophosphamide ifosfamide, darcarbazine). As a chemopotentiator, RSU-1069 might be clinically useful at low doses that avoid the previously described severe side-effects.

This research was supported in part by grants CA-44106 (K.T.W.) and CA-49498 (C.J.K.) from the National Cancer Institute, National Institutes of Health and in part by BRS grant RR-05404 awarded to the Bowman Gray School of Medicine from the National Institutes of Health. The authors thank N.R. Lomax of the Drug Synthesis and Chemistry Branch, Division of Cancer Treatment, National Cancer Institute and Dr M. Suto at Warner-Lambert for supplying the RSU-1069. We also thank Dr T.M. Morgan from the Biostatistics Unit of the Wake Forest University Cancer Center for help with the statistical analyses and J. Cornelius and D. Cantrell for preparation of this manuscript.

### References

ADAMS, G.E., AHMED, I., SHELDON, P.W. & STRATFORD, I.J. (1984*a*). Radiation sensitization and chemopotentiation: RSU-1069, a compound more efficient than misonidazole *in vitro* and *in vivo*. *Br. J. Cancer*, **49**, 57.

ADAMS, G.E., AHMED, I., SHELDON, P.W. & STRATFORD, I.J. (1984*b*). RSU-1069, a 2-nitroimidazole containing an alkylating group: high efficiency as a radio- and chemosensitizer *in vitro* and *in vivo*. *Int. J. Radiat. Oncol. Biol. Phys.*, **10**, 1653.

ADAMS, G.E., STRATFORD, I.J., WALLACE, R.G. & 2 others (1980). Toxicity of nitro compounds towards hypoxic mammalian cells *in vitro*: dependence upon reduction potential. *J. Natl Cancer Inst.*, **64**, 555.

AHMED, I., JENKINS, T.C., WALLING, J.M. & 4 others (1986). Analogs of RSU-1069: Radiosensitization and toxicity *in vitro* and *in vivo*. *Int. J. Radiat. Oncol. Biol. Phys.*, **12**, 1079.

BREMNER, J.C.M., STRATFORD, I.J., BOWLER, J. & ADAMS, G.E. (1990). Bioreductive drugs and the selective induction of tumour hypoxia. *Br. J. Cancer*, **61**, 717.

BROWN, J.M. (1982). The mechanisms of cytotoxicity and chemopotentiation by misonidazole and other nitroimidazoles. *Int. J. Radiat. Oncol. Biol. Phys.*, **8**, 675.

CHAPLIN, D.J., DURAND, R.E., STRATFORD, I.J. & JENKINS, T.C. (1986). The radiosensitizing and toxic effects of RSU-1069 on hypoxic cells in a murine tumour. *Int. J. Radiat. Oncol. Biol. Phys.*, **12**, 1091.

CLARK, E.P., WARDMAN, P. & GOULDING, K.H. (1980). Anaerobic reduction of nitroimidazoles by reduced flavin mononucleotide by xanthine oxidase. *Biochem. Pharmacol.*, **29**, 2684.

COLE, S., STRATFORD, I.J. & ADAMS, G.E. (1989). Manipulation of radiological hypoxia in a human melanoma xenograft to exploit the bioreductive cytotoxicity of RSU-1069. *Int. J. Radiat. Biol.*, **56**, 587.

DEACON, J.M., HOLLIDAY, S.B., AHMED, I. & JENKINS, T.C. (1986). Experimental pharmacokinetics of RSU-1069 and its analogues: high tumor/plasma ratio. *Int. J. Radiat. Oncol. Biol. Phys.*, **12**, 1087.

HORWICH, A., HOLLIDAY, S.B., DEACON, J.M. & PECKHAM, M.J. (1986). A toxicity and pharmacokinetic study in man of the hypoxic-cell radiosensitizer RSU-1069. *Br. J. Radiol.*, **59**, 1238.

KEOHANE, A., GODDEN, J., STRATFORD, I.J. & ADAMS, G.E. (1990). The effects of three bioreductive drugs (mitomycin C, RSU-1069 and SR4233) on cell lines selected for their sensitivity to mitomycin C or ionizing radiation. *Br. J. Cancer*, **61**, 722.

KOCH, C.J. (1984). A thin-film culturing technique allowing rapid gas-liquid equilibration (6 sec) with no toxicity to mammalian cells. *Radiat. Res.*, **97**, 434.

KOCH, C.J. & PAINTER, R.B. (1975). The effect of extreme hypoxia on the repair of DNA single-strand breaks in mammalian cells. *Radiat. Res.*, **64**, 256.

KOCH, C.J., STOBBE, C.C. & BUMP, E.A. (1984). The effect on $K_m$ for radiosensitization at 0°C of thiol depletion by diethylmaleate pretreatment: Quantitative differences found using the radiation sensitizing agents misonidazole and oxygen. *Radiat. Res.*, **98**, 141.

OLIVE, P.L., DURAND, R.E. & CHAPLIN, D.J. (1987). Cytotoxicity of RSU-1069 in spheroids and murine tumours. *Int. J. Radiat. Oncol. Biol. Phys.*, **13**, 1361.

ROSENBLUM, M.L., KNEBEL, K.D., VASQUES, D.A. & WILSON, C.B. (1976). *In vivo* clonogenic tumor cell kinetics following 1,3-bis(2-chloroethyl)-1-nitrosourea brain tumor therapy. *Cancer Res.*, **36**, 3718.

SIEMANN, D.W. (1989). The chemosensitizing and cytotoxic effects of RSU-1164 and RSU-1165 in a murine tumor model. *Int. J. Radiat. Oncol. Biol. Phys.*, **16**, 1115.

SIEMANN, D.W., ALLIET, K., MADDISON, K. & WOLF, K. (1985). Enhancement of the antitumour-efficacy of lomustine by the radiosensitizer RSU-1069. *Cancer Treat. Rep.*, **69**, 1409.

STRATFORD, I.J., WALLING, J.M. & SILVER, A.R.J. (1986). The differential cytotoxicity of RSU-1069: cell survival studies indicating interaction with DNA as a possible mode of action. *Br. J. Cancer*, **53**, 339.

WALLEN, C.A., MICHAELSON, S.M. & WHEELER, K.T. (1980). Evidence of an unconventional radiosensitivity of 9L subcutaneous tumors. *Radiat. Res.*, **84**, 529.

WALLING, J.M., DEACON, J., HOLLIDAY, S. & STRATFORD, I.J. (1989). High uptake of RSU-1069 and its analogues into melanotic melanomas: a cell mediated event. *Cancer Chemother. Pharmacol.*, **24**, 28.

WALTON, M.I. & WORKMAN, P. (1988). Pharmacokinetics and metabolism of the mixed-function hypoxic cell sensitizer prototype, RSU-1069, in mice. *Cancer Chemother. Pharmacol.*, **22**, 275.

WHEELER, K.T., WALLEN, C.A., WOLF, K.L. & SIEMANN, D.W. (1984). Hypoxic cells and *in situ* chemopotentiation of the nitrosoureas by misonidazole. *Br. J. Cancer*, **49**, 787.

WHITMORE, G.F. & GULYAS, S. (1986). Studies on the toxicity of RSU-1069. *Int. J. Radiat. Oncol. Biol. Phys.*, **12**, 1219.

WONG, K-H., WALLEN, C.A. & WHEELER, K.T. (1989). Biodistribution of misonidazole and 1,3-bis(2-chloroethyl)-1-nitrosourea (BCNU) in rats bearing unclamped and clamped 9L subcutaneous tumors. *Int. J. Radiat. Oncol. Biol. Phys.*, **17**, 135.

WONG, K-H., WALLEN, C.A. & WHEELER, K.T. (1990). Chemosensitization of the nitrosoureas by 2-nitroimidazoles in the subcutaneous 9L tumour model: pharmacokinetic and structure-activity considerations. *Int. J. Radiat. Oncol. Biol. Phys.*, **18**, 1043.